# World orofacial motricity day – a decade reaffirming the role of the speech-language pathologist in the assessment and treatment of orofacial functions

**DOI:** 10.1590/2317-1782/e20250067en

**Published:** 2025-11-17

**Authors:** Lucas Ferreira, Giédre Berretin-Felix, Roberta Lopes de Castro Martinelli, Giorvan Ânderson dos Santos Alves, Viviane Veroni Degan, Gabriele Ramos de Luccas, Gislaine Aparecida Folha, Hilton Justino da Silva

**Affiliations:** 1 Programa de Pós-graduação em Saúde da Comunicação Humana – PPGSCH, Universidade Federal de Pernambuco – UFPE - Recife (PE), Brasil.; 2 Departamento de Fonoaudiologia, Faculdade de Odontologia de Bauru – FOB, Universidade de São Paulo – USP - Bauru (SP), Brasil.; 3 Associação Brasileira de Motricidade Orofacial – ABRAMO - São Paulo (SP), Brasil.; 4 Hospital Santa Therezinha - Brotas (SP), Brasil.; 5 Programa Associado de Pós-graduação em Fonoaudiologia, Departamento de Fonoaudiologia, Universidade Federal da Paraíba – UFPB - João Pessoa (PB), Brasil.; 6 Departamento de Motricidade Orofacial, Sociedade Brasileira de Fonoaudiologia – SBFa - São Paulo (SP), Brasil.; 7 Departamento de Ciências da Saúde, Faculdade de Medicina de Ribeirão Preto – FMRP, Universidade de São Paulo – USP - Ribeirão Preto (SP), Brasil.; 8 Programa de Pós-graduação em Saúde da Comunicação Humana, Departamento de Fonoaudiologia, Universidade Federal de Pernambuco – UFPE - Recife (PE), Brasil.

## APAGAR

Dear Editors-in-Chief of CoDAS,

It is with great pleasure that we write this letter to address a date of significant importance within the field of Speech-Language Pathology. Our aim is to highlight key milestones in the area of Orofacial Myology (OM) over the past 10 years, as well as to reflect on the themes that have shaped the history of World OM Day.

It is important to note that OM is dedicated to the study, research, development, diagnosis, and rehabilitation of congenital or acquired disorders affecting the orofacial and cervical myofunctional system. These conditions may arise from gestation through aging, and professional practice in this field seeks to restore the functionality and harmony of essential orofacial functions, such as sucking, breathing, swallowing, chewing, and speech^(1-3)^.

Currently, Brazil has 2,006 (data from September 2025) specialists in OM registered with the Federal Council of Speech-Language Pathology (CFFa), making it the second largest specialty in number of professionals in the country. However, this number may be even higher, considering that many speech-language pathologists work in the field without necessarily holding the specialist title^(4)^.

The history of OM in Brazil dates back to the 1970s, but it was only from the 1980s onward that scientific production increased significantly, with the publication of books, book chapters, articles, dissertations, and theses^(5-7)^. In 1998, with the creation of the Orofacial Myology Committee—currently known as the Orofacial Myology Department of the Brazilian Society of Speech-Language Pathology (SBFa), the field was officially consolidated within the scientific community^(4,5)^. A key milestone occurred on February 17, 2006, with the publication of CFFa Resolution No. 320, which recognized Orofacial Myology as a specialty, formalizing its integration into the context of Speech-Language Pathology in Brazil^(1)^. In 2015, the legalization of the Brazilian Association of Orofacial Myology (ABRAMO) further strengthened the specialty, highlighting its relevance for professional and scientific development in the country^(8,9)^.

Concomitantly, the specialty has gained strength in scientific events with the growing inclusion of topics related to orofacial functions in congresses and symposia. The Brazilian Meeting of Orofacial Myology (EBMO), held for the past 17 years, has promoted and strengthened research and technical-scientific discussions in the field, reinforcing its relevance within Speech-Language Pathology. Another noteworthy event is the Brazilian Congress of Speech-Language Pathology (CBFa), which has dedicated a specific room for advanced discussions on OM, addressing innovative therapies and updates of clinical protocols. In addition, OM is also represented in other thematic sessions, such as those on infant feeding and its disorders, highlighting its intersections with different specialties.

**Figure 1:**
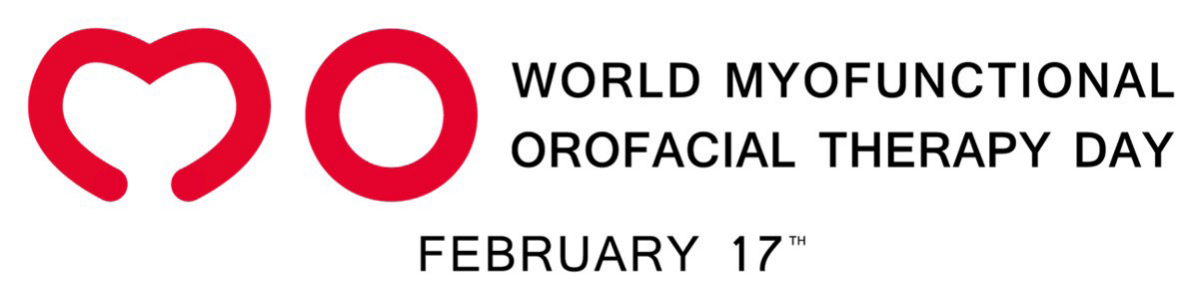
Official logo for World Orofacial Myology Day Orofacial

Building on this growth and the increasing relevance of the field, on June 26, 2015, the *“II Encuentro Americano y I Iberoamericano de Motricidad Orofacial”* was held in Lima, Peru, with the participation of representatives from Argentina, Brazil, Chile, Colombia, Ecuador, Spain, the United States, Mexico, Panama, Peru, and Portugal. During the meeting, the “World Orofacial Myology Day” was officially established, with representatives from each country signing the commitment, and Dr. Irene Queiroz Marchesan serving as Brazil’s representative^(9)^. The official logo for this commemorative date, created by the Instituto EPAP (Portugal) in partnership with CEFAC (an educational institution), is shown in Figure 1.

Celebrated annually, World Orofacial Myology Day highlights the importance of orofacial functions for health and well-being, as well as the role of speech-language pathologists as professionals qualified to assess and intervene in disorders related to these functions and to foster their improvement across different stages of life. This event, which celebrates the achievements of the field, has become established over the past decade with slogans addressing a wide range of OM issues, raising awareness and encouraging progress in professional practice. Each year, a central theme is proposed to guide the activities developed for the celebration, chosen according to current demands and the most prominent topics in OM during that period.

Additionally, it is paramount to remember that on February 17 we celebrate the birthday of Dr. Irene Queiroz Marchesan, a pioneer of OM in Brazil and the first speech-language pathologist to receive the title of specialist in this area from the CFFa^(4)^. The date was chosen in her honor, as her work was essential for the development and consolidation of this specialty both in Brazil and internationally.

Over the past decade, several themes have been defined for World Orofacial Myology Day, always emphasizing that the assessment and therapy of orofacial functions fall within the scope of speech-language pathologists. This date has consistently received the support and participation of SBFa, through its OM Department, ABRAMO, and other scientific entities from Latin America and Europe. Chart 1 presents the slogans from the past 10 years.

**Chart 1:** Slogans for World Myology Day in the last decade

● **2016**: “Breathing: have you stopped to think about it?”
● **2017**: “Tongue-tie, disturbed orofacial functions”
● **2018**: “Let´s talk about speech”
● **2019**: “Do you think chewing well is important? Why?”
● **2020**: “Oral habits in the childhood – from prevention to treatment”
● **2021**: “COVID-19, breathing and swallowing problems”
● **2022**: “Assessment and treatment of orofacial functions are up to speech-language pathologists ”
● **2023**: “Orofacial functions: how to prevent orofacial myofunctional disorders?”
● **2024**: “The role of speech-language pathologists in breathing and sleeping disorders”
● **2025**: “The impact of tongue-tie on breathing, feeding and speech. How speech-language pathologists can contribute?”

**Source:** Authors elaboration

In recent years, World Orofacial Myology Day has addressed essential themes for orofacial health. In 2016, the first theme focused on breathing and its importance for the balance of the respiratory system. In the following years, the focus turned to the impacts of tongue-tie (2017), speech (2018), and chewing (2019), underscoring the need for proper assessment and intervention. In 2020, the emphasis was placed on preventing harmful oral habits in childhood, highlighting the importance of early intervention and the role of speech-language pathologists in diagnosis, in collaboration with other professionals, such as those in Dentistry.

The COVID-19 pandemic (2021) posed challenges for breathing and swallowing, particularly due to the sequelae of infection and the length of orotracheal intubation, in addition to disturbances of smell and taste, underscoring the importance of specialized follow-up by speech-language pathologists. In 2022, the role of these professionals in the diagnosis and treatment of orofacial functions was reaffirmed, followed in 2023 by a focus on preventive approaches to orofacial functions throughout life. In 2024, the theme centered on the relationship between “breathing and sleep disorders,” emphasizing how respiratory disturbances can be prevented and managed with speech-language pathology support, while reaffirming the relevance of areas such as sleep certification within the field. For 2025, the theme returns to “tongue-tie” (ankyloglossia), this time exploring its impacts on breathing, feeding, and speech, and highlighting the central role of speech-language pathologists in diagnosis, prevention, and treatment. In the coming years, other themes will be addressed, with the main focus on promoting awareness of OM practice as well as its role within interdisciplinary teams.

Throughout this decade, the themes chosen for World Orofacial Myology Day have reflected significant advances in the understanding and management of orofacial functions, highlighting the essential role of speech-language pathologists in promoting orofacial health. Furthermore, the celebration reinforces the recognition of the pioneering work of Dr. Irene Queiroz Marchesan, whose contribution was decisive for consolidating the specialty in Brazil and for training professionals qualified to practice based on scientific knowledge and updated clinical approaches.

In addition to promoting recognition of the specialty, World Orofacial Myology Day has had a direct impact on clinical and healthcare practice. There has been a noticeable increase in public awareness of orofacial myofunctional disorders, leading to greater demand for specialized care. Speech-language pathologists report growing demand for early assessments and evidence-based interventions. This growth has been documented in studies that highlight the expansion and consolidation of publications^(10,11)^ in the field of OM over the past decade. Nevertheless, further progress is still needed, which may be driven by the efforts of scientific institutions as well as academic and research programs in Speech-Language Pathology.

Research in OM has therefore expanded significantly, with notable advances in the understanding of the neurophysiology of orofacial functions, the development of new assessment and treatment techniques, and the use of innovative technologies. Approximately half of the national publications in the field make use of some type of technology, with semi-hard technologies being the most frequent, followed by hard and soft ones^(12)^. The incorporation of these technologies reflects the growing sophistication of diagnostic and rehabilitation methods.

However, for OM to continue evolving, it is essential to maintain consistent investment in the development of national and international evidence-based guidelines, terminological standardization, and validation of clinical protocols, including translations and cross-cultural adaptations that broaden their global applicability. The design and implementation of health prevention campaigns and initiatives should also be encouraged, especially to promote early diagnosis and intervention. Establishing consensus among researchers and clinicians, alongside fostering multicenter studies and incorporating new technologies, will strengthen the field, ensuring greater diagnostic accuracy, therapeutic effectiveness, and impact on public health.
