# Supplementary material for: World orofacial motricity day – a decade reaffirming the role of the speech-language pathologist in the assessment and treatment of orofacial functions
Source: Codas. 2025 Nov 17;37(6):e20250067. doi: 10.1590/2317-1782/e20250067en (PMC12699483; doi:10.1590/2317-1782/e20250067en)
Supplement: Translation

# Translation: Dia mundial da motricidade orofacial – uma década reafirmando o papel do fonoaudiólogo na avaliação e tratamento das funções orofaciais

**DOI:** 10.1590/2317-1782/e20250067pt

**Authors:** Lucas Ferreira, Giédre Berretin-Felix, Roberta Lopes de Castro Martinelli, Giorvan Ânderson dos Santos Alves, Viviane Veroni Degan, Gabriele Ramos de Luccas, Gislaine Aparecida Folha, Hilton Justino da Silva

**Affiliations:** 1 Programa de Pós-graduação em Saúde da Comunicação Humana – PPGSCH, Universidade Federal de Pernambuco – UFPE - Recife (PE), Brasil.; 2 Departamento de Fonoaudiologia, Faculdade de Odontologia de Bauru – FOB, Universidade de São Paulo – USP - Bauru (SP), Brasil.; 3 Associação Brasileira de Motricidade Orofacial – ABRAMO - São Paulo (SP), Brasil.; 4 Hospital Santa Therezinha - Brotas (SP), Brasil.; 5 Programa Associado de Pós-graduação em Fonoaudiologia, Departamento de Fonoaudiologia, Universidade Federal da Paraíba – UFPB - João Pessoa (PB), Brasil.; 6 Departamento de Motricidade Orofacial, Sociedade Brasileira de Fonoaudiologia – SBFa - São Paulo (SP), Brasil.; 7 Departamento de Ciências da Saúde, Faculdade de Medicina de Ribeirão Preto – FMRP, Universidade de São Paulo – USP - Ribeirão Preto (SP), Brasil.; 8 Programa de Pós-graduação em Saúde da Comunicação Humana, Departamento de Fonoaudiologia, Universidade Federal de Pernambuco – UFPE - Recife (PE), Brasil.

Prezados Editores-Chefes da Revista CoDAS,

É com grande satisfação que redigimos esta carta para abordar uma data de significativa importância dentro da Fonoaudiologia. Nosso objetivo é destacar alguns marcos relevantes para a área da Motricidade Orofacial (MO) nos últimos 10 anos, além de refletir sobre os temas que têm construído a história do Dia Mundial da MO.

É relevante destacar que a MO se dedica ao estudo, pesquisa, aperfeiçoamento, diagnóstico e reabilitação de transtornos congênitos ou adquiridos que afetam o sistema miofuncional orofacial e cervical. Essas condições podem surgir desde a gestação até o envelhecimento, e a atuação na área tem como objetivo restaurar a funcionalidade e harmonia das funções orofaciais essenciais, como sucção, respiração, deglutição, mastigação e fala^(1-3)^.

Atualmente, o Brasil conta com 2.006 (dados referentes a setembro de 2025) especialistas em MO registrados no Conselho Federal de Fonoaudiologia (CFFa), tornando a segunda maior especialidade em número de profissionais no país. No entanto, esse número pode ser ainda maior, considerando que muitos fonoaudiólogos atuam na área sem necessariamente possuírem o título de especialista^(4)^.

A história da MO no Brasil remonta à década de 70, e somente a partir de 1980, houve um aumento significativo na produção científica, com a publicação de livros, capítulos, artigos, dissertações e teses^(5-7)^. Em 1998, com a criação do Comitê de Motricidade Orofacial, atualmente denominado Departamento de Motricidade Orofacial da Sociedade Brasileira de Fonoaudiologia (SBFa), a área foi oficialmente consolidada dentro da entidade científica^(4,5)^. Um marco fundamental ocorreu em 17 de fevereiro de 2006, com a publicação da Resolução CFFa nº 320, que reconheceu a especialidade de Motricidade Orofacial, formalizando sua integração ao contexto da Fonoaudiologia no Brasil^(1)^. Em 2015, a legalização da Associação Brasileira de Motricidade Orofacial (ABRAMO) fortaleceu ainda mais a especialidade, destacando sua relevância para o desenvolvimento profissional e científico no país^(8,9)^.

Concomitantemente, a especialidade se fortaleceu nos eventos científicos com a crescente inclusão de temas relacionados às funções orofaciais em congressos e simpósios. O Encontro Brasileiro de Motricidade Orofacial (EBMO), há 17 anos, promove e fortalece as pesquisas e discussões técnico-científicas na área, reforçando sua relevância dentro da Fonoaudiologia. Outro evento de destaque é o Congresso Brasileiro de Fonoaudiologia (CBFa), que tem dedicado uma sala específica para discussões avançadas sobre MO, abordando terapias inovadoras e a atualização de protocolos clínicos. Além disso, a área de MO também se faz presente em outras salas temáticas, como a de alimentação infantil e seus distúrbios, evidenciando suas interfaces com diferentes especialidades.

A partir desse crescimento e da relevância da área que no dia 26 de junho de 2015, realizou-se na cidade de Lima, no Peru, o “*II Encuentro Americano y I Iberoamericano de Motricidad Orofacial*”, evento que contou com a participação de representantes da Argentina, Brasil, Chile, Colômbia, Equador, Espanha, Estados Unidos, México, Panamá, Peru e Portugal. Durante o encontro, foi criado oficialmente o “Dia Mundial da Motricidade Orofacial”, com a assinatura do compromisso dos representantes de cada país, sendo a Dra. Irene Queiroz Marchesan a representante do Brasil^(9)^. O logotipo oficial dessa data comemorativa, criado pelo Instituto EPAP (Portugal) em parceria com o CEFAC, está representado na Figura 1.

**Figura 1:** Logotipo oficial do Dia Mundial da Motricidade Orofacial

Comemorado anualmente, o Dia Mundial da MO ressalta a importância das funções orofaciais para a saúde e o bem-estar, bem como a relevância do fonoaudiólogo como profissional apto a avaliar e intervir nos distúrbios relacionados a essas funções e no seu aperfeiçoamento nos diferentes ciclos de vida. Este evento, que celebra as conquistas da área, tem se consolidado ao longo da última década, com slogans que abordam as diversas questões sobre a MO, promovendo a conscientização e incentivando avanços na prática profissional. Em cada ano é proposto um tema principal que guia as atividades elaboradas em comemoração, sendo escolhido a partir das demandas atuais e em maior destaque na MO naquele período.

Adicionalmente, é significativo lembrarmos que, no dia 17 de fevereiro, celebramos o aniversário da Dra. Irene Queiroz Marchesan, pioneira na MO no Brasil e a primeira fonoaudióloga a receber o título de especialista nesta área pelo CFFa^(4)^. A data foi escolhida em sua homenagem, pois seu trabalho foi essencial para o desenvolvimento e a consolidação dessa especialidade, tanto no Brasil quanto internacionalmente.

Ao longo da última década, diversos temas foram definidos para o Dia Mundial da MO, sempre destacando a importância de que a avaliação e a terapia das funções orofaciais são de competência do fonoaudiólogo. Esta data sempre teve apoio e participação da SBFa, por meio do Departamento de MO, da ABRAMO entre outras entidades científicas da América Latina e Europa. A seguir, apresentamos no Quadro 1 os slogans ao longo dos 10 anos.

**Quadro 1:** Slogans do Dia Mundial da Motricidade Orofacial na última década

● **2016**: “Respirar: já parou para pensar?”
● **2017**: “Língua presa, funções orofaciais prejudicadas”
● **2018**: “Falemos da Fala”
● **2019**: “Você acha que mastigar bem é importante? Por quê?”
● **2020**: “Hábitos orais na primeira infância – da prevenção ao tratamento”
● **2021**: “COVID-19, problemas na Respiração e Deglutição”
● **2022**: “Avaliação e tratamento das funções orofaciais são com o fonoaudiólogo”
● **2023**: “Funções orofaciais: como prevenir alterações da Motricidade Orofacial?”
● **2024**: “Importância do papel do fonoaudiólogo nas alterações da respiração e do sono”
● **2025**: “O impacto da “língua presa” na respiração, alimentação e fala. Como o fonoaudiólogo pode contribuir?”

**Fonte:** Elaboração própria dos autores

Nos últimos anos, o Dia Mundial da MO abordou temas essenciais para a saúde orofacial. Em 2016, o primeiro tema trouxe como foco a respiração e sua importância para o equilíbrio do sistema respiratório. Nos anos seguintes, foram discutidos os impactos da língua presa (2017), a fala (2018) e a mastigação (2019), ressaltando a necessidade de avaliação e intervenções adequadas. Em 2020, a ênfase esteve na prevenção de hábitos orais inadequados na infância, destacando a importância da intervenção precoce e do papel do fonoaudiólogo nesse diagnóstico junto de outras profissões, como a área da Odontologia.

A pandemia de COVID-19 (2021) gerou desafios para a respiração e a deglutição, sobretudo devido às sequelas da infecção e ao tempo de intubação orotraqueal, além de distúrbios de olfato e paladar, ressaltando a importância do acompanhamento especializado pelo fonoaudiólogo. Em 2022, o papel deste profissional no diagnóstico e tratamento das funções orofaciais foi reafirmado, seguido, em 2023, por uma abordagem preventiva das funções orofaciais ao longo da vida. Em 2024, o foco foi na relação entre “alterações na respiração e o sono”, ressaltando como os distúrbios respiratórios podem ser prevenidos e tratados com apoio fonoaudiológico, reafirmando a relevância, por exemplo, da certificação do sono dentro da área. Para 2025, a discussão se volta novamente para a “língua presa” (anquiloglossia), agora explorando seus impactos na respiração, alimentação e fala, destacando o papel primordial deste profissional no diagnóstico, prevenção e tratamento. Para os próximos anos, outros temas serão abordados, tendo como principal foco a divulgação da atuação em MO, bem como o papel em equipes interdisciplinares.

Ao longo dessa década, os temas abordados no Dia Mundial da MO refletem avanços significativos na compreensão e manejo das funções orofaciais, destacando o papel essencial do fonoaudiólogo na promoção da saúde orofacial. Além disso, a celebração reforça o reconhecimento do trabalho pioneiro de Dra. Irene Queiroz Marchesan, cuja contribuição foi determinante para a consolidação da especialidade no Brasil e para a formação de profissionais capacitados a atuar com base em conhecimento científico e práticas atualizadas.

Além de promover o reconhecimento da especialidade, o Dia Mundial da MO tem impactado diretamente a prática clínica e assistencial. Observa-se um aumento na conscientização da população sobre os transtornos miofuncionais orofaciais, o que tem levado a uma maior busca por atendimento especializado. Profissionais da Fonoaudiologia relatam um crescimento na demanda por avaliações precoces e intervenções baseadas em evidências. Esse crescimento tem sido relatado em estudos que evidenciam a expansão e a consolidação das publicações^(10,11)^ na área de MO na última década, embora ainda sejam necessários avanços, os quais podem ser impulsionados pelo esforço das instituições científicas, bem como, de ensino e pesquisa em Fonoaudiologia.

Com isso a pesquisa na área de MO se expandiu significativamente, com avanços notáveis na compreensão da neurofisiologia das funções orofaciais, no desenvolvimento de novas técnicas de avaliação e tratamento, bem como no uso de tecnologias inovadoras. Aproximadamente metade das publicações nacionais na área faz uso de alguma tecnologia dessas ferramentas, sendo as tecnologias leve-dura as mais frequentes, seguidas pelas duras e leves^(12)^. A incorporação dessas tecnologias reflete a crescente sofisticação dos métodos de diagnóstico e reabilitação.

Porém, para a contínua evolução da MO, é fundamental o constante investimento na consolidação de diretrizes nacionais e internacionais baseadas em evidências, padronização terminológica e validação de protocolos clínicos, com traduções e adaptações transculturais que ampliem sua aplicabilidade global. A elaboração e vinculação de campanhas e ações de prevenção em saúde também devem ser encorajadas, principalmente para estimular diagnósticos e intervenções precoces. O estabelecimento de consensos entre pesquisadores e clínicos, aliado ao incentivo a estudos multicêntricos e à incorporação de novas tecnologias, fortalecerá a área, garantindo maior precisão diagnóstica, eficácia terapêutica e impacto na saúde pública.
